# Supplementary material for: Application of Nanopore Sequencing (MinION) for the Analysis of Bacteriome and Resistome of Bean Sprouts
Source: Microorganisms. 2021 Apr 27;9(5):937. doi: 10.3390/microorganisms9050937 (PMC8146283; doi:10.3390/microorganisms9050937)
Supplement: Supplementary file 1 [file microorganisms-09-00937-s001.zip › microorganisms-1187544-supplementary.pdf]

Table S1. Family diversity of bean sprouts – other fourteen abundant families (detected only from <0.5 % to 4 %)

Family/ Genus	Sample A (0 h)<br>**12 441**	Sample A (48 h)<br>**209 702**	Sample B (0 h)<br>**13 728**	Sample B (48 h)<br>**378 082**
***Leuconostocaceae* (G+)**	**0 (0 %)**	**6 (<0.5 %)**	**171 (2 %)**	**13 170 (4 %)**
Leuconostoc	0 %	<0.5%	<0.5%	1%
Weissella	0 %	<0.5%	1%	3%
***Pectobacteriaceae* (G-)**	**3 (<0.5 %)**	**307 (<0.5 %)**	**30 (<0.5 %)**	**8 407 (2 %)**
Pectobacterium	0 %	<0.5%	<0.5%	2%
Dickeya	<0.5 %	<0.5%	<0.5%	<0.5%
***Yersiniaceae* (G-)**	**1 (<0.5 %)**	**411 (<0.5 %)**	**98 (1 %)**	**5 577 (2 %)**
Yersinia	0 %	<0.5%	<0.5%	<0.5%
Serratia	<0.5 %	<0.5%	1%	1%
***Comamonadaceae* (G-)**	**1 (<0.5 %)**	**4 764 (2 %)**	**7 (<0.5 %)**	**5 259 (1 %)**
Comamonas	<0.5 %	1%	<0.5%	1%
Delftia	0 %	1%	<0.5%	<0.5%
***Campylobacteriaceae* (G-)**	**1 (<0.5 %)**	**3 604 (2 %)**	**4 (<0.5 %)**	**56 (<0.5 %)**
Campylobacter	<0.5 %	<0.5%	<0.5%	<0.5%
Arcobacter	0 %	2%	<0.5%	<0.5%
***Aeromonadaceae* (G-)**	0 (0 %)	**808 (<0.5 %)**	**2 (<0.5 %)**	**3 968 (1 %)**
Aeromonas	0 %	<0.5%	<0.5%	1%
***Chromobacteriaceae* (G-)**	**1 (<0.5 %)**	**844 (<0.5 %)**	**2 (<0.5 %)**	**166 (<0.5 %)**
Chromobacterium	0 %	<0.5%	0%	<0.5%
Laribacter	0 %	<0.5%	<0.5%	<0.5%
***Burkholderiaceae* (G-)**	**2 (<0.5 %)**	**1 020 (1 %)**	**8 (<0.5 %)**	**1 125 (<0.5 %)**
Burkholderia	<0.5 %	<0.5%	<0.5%	<0.5%
Cupriavidus	<0.5 %	<0.5%	<0.5%	<0.5%
***Xanthomonadaceae* (G-)**	**2 (<0.5 %)**	**542 (<0.5 %)**	**4 (<0.5 %)**	**991 (<0.5 %)**
Xanthomonas	<0.5 %	<0.5%	0%	<0.5%
Lysobacter	0 %	<0.5%	<0.5%	<0.5%
***Neisseriaceae* (G-)**	**0 (<0.5 %)**	**34 (<0.5 %)**	**0 (<0.5 %)**	**41 (<0.5 %)**
Neisseria	0 %	<0.5%	0%	<0.5%
***Enterococcaceae* (G+)**	**1 (<0.5 %)**	**325 (<0.5 %)**	**6 (<0.5 %)**	**1 409 (<0.5 %)**

Enterococcus	0 %	<0.5%	<0.5%	<0.5%
Vagococcus	0 %	<0.5%	<0.5%	<0.5%
***Morganellaceae* (G-)**	**1 (<0.5 %)**	**68 (<0.5 %)**	**5 (<0.5 %)**	**132 (<0.5 %)**
Morganella	0 %	<0.5%	0%	<0.5%
Proteus	0 %	<0.5%	<0.5%	<0.5%
Providencia	0 %	<0.5%	<0.5%	<0.5%
***Lactobacillaceae* (G+)**	**2 (<0.5 %)**	**89 (<0.5 %)**	**5 (<0.5 %)**	**427 (<0.5 %)**
Lactobacillus	<0.5 %	<0.5%	<0.5%	<0.5%
Pediococcus	<0.5%	<0.5%	0%	<0.5%
***Hafniaceae* (G-)**	**1 (<0.5 %)**	**147 (<0.5 %)**	**7 (<0.5 %)**	**136 (<0.5 %)**
Hafnia	0%	<0.5%	<0.5%	<0.5%
**Others families**	1%	3%	2%	3%
